# Liquid Biopsy Meets Assembloids: PSA/miRNA/IL in Prostate Cancer

**DOI:** 10.3390/cancers18142274

**Published:** 2026-07-15

**Authors:** Joanna Bialek, Theresa Rohr, Paolo Fornara, Karl Weigand, Georgios Gakis, Gerit Theil

**Affiliations:** 1 Medical Faculty of Martin Luther University Halle-Wittenberg, University Clinic and Outpatient Clinic for Urology, 06120 Halle (Saale), Germany; rohr.theresa@gmail.com (T.R.); paolo.fornara@uk-halle.de (P.F.); georgios.gakis@uk-halle.de (G.G.); gerit.theil@uk-halle.de (G.T.); 2 Department of Urology, University Medicine Rostock, 18057 Rostock, Germany; karl.weigand@med.uni-rostock.de

**Keywords:** prostate, PCa, microRNA, miR, prostate-specific antigen, PSA, liquid biopsy, LB, assembloids

## Abstract

**Background**: Clinical examination of prostate disorders includes PSA testing, which is organ- but not cancer-specific. MicroRNAs (miRs) contribute to pathological and physiological conditions of many organs. This study investigated PSA, miRs, and interleukins (ILs) and validated findings using prostate cell line-based assembloids. **Methods**: During a 10-year PSA-based observational study, the expression of miRs (-16, -141, -221, -222, and -375), IL-6, and IL-8 was analyzed in an exploratory cohort using digital PCR and chemiluminescent immunoassays. Prostate assembloids composed of epithelial and fibroblastic cell lines combined with healthy, BPH, or PCa cells were used for in vitro validation. Statistical analyses were performed using Kruskal–Wallis or ANOVA tests and ROC curve analysis. **Results**: We diagnosed PCa in 19 and BPH in 77 men. PSA and IL-8 levels significantly distinguished PCa from BPH and healthy controls. Serum miR-141 and -222 were reduced in BPH and slightly lower in the PCa group than in the healthy controls. PCa assembloids showed reduced miR-141 and elevated IL-6 and IL-8. ROC analysis showed that miR-141 and miR-222 discriminated BPH from healthy controls (AUCs 0.846 and 0.807), comparable to PSA, whereas PSA performed best for PCa. **Conclusions**: Our data suggest that miR-141 and miR-222 may complement PSA in prostate disorder diagnostics, showing comparable performance for BPH vs. healthy controls, while PSA performed best for PCa detection. However, given the limited sample size and the lack of external validation, our study should be considered exploratory and requires validation in larger, independent cohorts.

## Simple Summary

Prostate cancer (PCa) was the most frequently diagnosed cancer in men in Germany in 2022. Biopsy, as the standard testing method, is invasive and painful, increases the risk of infection, and yields inaccurate or underestimated staging results in approximately one-third of cases. An alternative is minimally invasive liquid biopsy (LB). The aim of the 10-year observational study was to detect changes in prostate-specific antigen (PSA) levels in serum samples and compare them with diagnostic outcomes. Furthermore, in an explorative cohort, we investigated whether combining additional factors like free PSA (fPSA), microRNAs (miRs), and interleukins in serum with PSA could improve diagnostic performance for PCa. We introduced the generation of prostate assembloids with defined cell compositions mimicking different prostate conditions in vitro. This allowed us to analyze and investigate factors in a controlled experimental model, within the broader context of cancer.

## 1. Introduction

According to GLOBOCAN 2022, PCa was the most frequently diagnosed cancer in men in Germany, with 65,269 new cancer cases, and was the second leading cause of cancer-related death in men in 2022 [1,2,3]. Early detection of PCa is crucial for effective treatment. Although localized prostate tumors are often asymptomatic and associated with favorable survival outcomes, a subset of patients may still develop metastatic disease with poor prognosis [3,4].

A biopsy is a routinely recommended medical intervention for suspected PCa, providing histopathological confirmation, Gleason score (GS), and clinical prognosis evaluation. Although biopsy is the standard testing method, it is invasive and painful, increases the risk of infection, and yields inaccurate or underestimated staging results in approximately one-third of cases [5].

An alternative is minimally invasive liquid biopsy (LB), which enables the analysis of components of body fluids, such as blood or urine. This technique can be used to detect circulating tumor cells (CTCs), cell-free RNA or DNA (cfRNA, cfDNA), proteins released from primary and metastatic tumors, and molecular alterations in body fluids induced directly by the tumor or in response to the tumor by surrounding tissues [5].

Initially, the clinical evaluation of prostate disorders in our studies relied on PSA testing. This surrogate marker can be included as a blood-based component of LB analysis. However, PSA is organ-specific but not cancer-specific, as elevated levels may also occur in benign conditions such as benign prostatic hyperplasia and prostatitis. To improve diagnostic specificity in men with total PSA concentrations between 3.8 and 10 ng/mL, the percentage of free PSA (%fPSA), calculated as the ratio of free to total PSA (fPSA/PSA × 100), is routinely determined. The free-to-total PSA ratio provides additional information for estimating the likelihood of PCa and supports biopsy decision-making in this patient group [6].

Chronic inflammation and immune signaling are increasingly recognized as important contributors to prostate cancer (PCa) development and progression [7]. Proinflammatory cytokines, particularly interleukin-6 (IL-6) and interleukin-8 (IL-8), have been implicated in tumor growth, angiogenesis, and disease progression [8]. Katongole et al. demonstrated an association between IL-6 and IL-8 with PSA in their cohort, suggesting their potential utility as biomarkers in PCa pathogenesis [9]. Elevated circulating IL-6 levels have been associated with advanced and metastatic PCa and with castration-resistant disease. Mechanistically, IL-6 activates oncogenic signaling pathways (e.g., JAK/STAT3 and PI3K/AKT signaling), thereby promoting tumor cell proliferation and inhibiting apoptosis [8]. Clinical studies have also reported correlations between IL-6, IL-8, and PSA levels, suggesting their potential utility as biomarkers linked to tumor burden and inflammatory activity [10].

Recently, the contribution of miRs to pathological and physiological conditions has been established. These small (approximately 18–25 nucleotides) noncoding RNA molecules regulate gene expression by binding to complementary sequences in target mRNAs, thereby inducing translational repression or degradation and affecting their activity and biological processes such as proliferation or differentiation [11]. Depending on the pathological or physiological conditions, one particular miR can play opposite roles in the same tissue because of the influence of the tissue microenvironment or 3’-untranslated region of different isoforms [12]. It can act as an oncogene or tumor suppressor [13]. In this way, the expression of up to 60% of protein-coding genes can be controlled [12].

The presence of circulating cell-free miRs provides the possibility to measure them in serum complementary to PSA within the framework of low-invasive liquid biopsy. Their stability and detectability depend on vesicular or non-vesicular release mechanisms. Encapsulated in secretory vesicles, they are stable and can be incorporated into the cell–cell communication process [14]. They can also be released into the circulation in a complex with proteins or lipid-associated carriers, which protect them from enzymatic digestion [15]. In adaptation to therapy, stressed cells can passively release miRs because of cell death [16]. The contribution of miRs to PCa development as factors expressed in tissues or soluble molecules has been postulated [17,18]. In 2008, Mitchell et al. [19] demonstrated the ability to measure miRs originating from PCa xenografts in mouse blood, establishing a proof-of-principle for blood-based miRNA biomarkers. Among the miRNAs investigated in PCa, miR-16 influences signaling pathways by targeting genes associated with PCa [20]. In patients, its expression in plasma was correlated with a high GS [21], whereas in other studies, decreased serum levels were reported in PCa patients compared with nonmalignant controls, with the opposite trend observed for clinicopathological parameters [22]. Similarly, circulating miR-141 and miR-375 (members of the miR-200 family and regulators of androgen pathways) are consistently associated with advanced and metastatic PCa. Analysis of the serum levels of miR-141 and miR-375 revealed differences between local and metastatic PCa samples, as well as between primary tissue samples and normal prostate tissue samples [8]. These two miRs are valuable markers for identifying high-risk tumors [23]. miR-222 and -221 are transcribed together as a gene cluster [24]. However, their roles in tumor progression are still unclear. Experimental studies with prostate carcinoma cell lines have reported miR-221 as an antiapoptotic factor [25] that supports epithelial–mesenchymal transition (EMT) [26]. Conversely, miR-221 acts as a tumor suppressor in an androgen-independent manner [27].

To better understand the effects of cell–cell interactions on protein or miR synthesis as well as their downstream consequences for tissue functionality, advanced 3D in vitro cell culture systems, such as assembloids, have been established. Assembloids are models of spatially organized spheroids that include a microenvironment and more closely mimic the in vivo tissue structures of solid tumors [28]. Through cell–cell contacts, allow interactions and, consequently, the regulation of signal transduction, differentiation, and secretion patterns [29]. Owing to their physiological features, such as the composition of normal, fibroblastic, and/or pathologic (tumor/hyperplastic) cell populations, they are potential models in tumor biology for mimicking disorder conditions and screening therapeutic responses [30].

We present data from a 10-year observational study on PCa enhanced with prostate assembloid analysis. Our primary aim was to detect potential changes in PSA levels in serum samples from participating men and the association of these changes with diagnosis. In an exploratory cohort including patients with PCa, patients with BPH, and healthy men, we investigated whether the combined assessment of fPSA, selected circulating miRs (miR-16, miR-141, miR-221, miR-222, and miR-375), and the interleukins IL6 and IL8 in serum could provide complementary diagnostic information beyond PSA alone. The generation of assembloids with defined compositions was established to model healthy, hyperplastic, and malignant prostate tissue and to investigate disease-associated molecular alterations in vitro.

## 2. Materials and Methods

### 2.1. Study Cohort and Blood Sampling

An observational study of PCa was performed for 10 years. Blood samples from men working for (or pensioners of) public utility companies were collected annually. Follow-up intervals for each subject were defined in agreement with the medical guidelines: a PSA result < 2 ng/mL—the next visit within 2–4 years; a PSA result > 2 ng/mL; and/or a family history of disease—the next visit at the latest in 1 year (depending on the PSA level). Only one visit was allowed for men younger than 40 years of age (the age recommended in the 2009 guidelines for early detection of PCa) who wanted to participate in the study [31]. Each of the 926 participants signed a consent form at every visit, allowing the use of their samples and anonymized clinical data for research purposes (4460 samples collected over the study period).

The biomarker analyses were performed in an exploratory case–control subgroup comprising 57 participants. This group included men who developed PCa during the observation period (*n* = 19), BPH (*n* = 19), and a control group of healthy men (*n* = 19) who were randomly selected from the age- and BMI-matched population without a family history of the disease.

Blood samples (4.5 mL) were collected in serum tubes and centrifuged at 9000 rpm for 10 min. Serum aliquots were frozen at −80 °C until further processing.

### 2.2. Cell Culture

The normal human prostate cell line RWPE-1 (CLS Cell Lines Service, Eppelheim, Germany) was cultivated in keratinocyte serum-free (KSF) medium supplemented with 0.05 mg/mL bovine pituitary extract, 5 ng/mL epidermal growth factor (Gibco, Thermo Fisher Scientific, Grand Island, NY, USA), and 10% FCS. The hyperplastic BPH-1 (Sigma-Aldrich, MilliporeSigma, Burlington, MA, USA) and carcinoma cell lines LNCaP and DU-145 were maintained in RPMI 1640 (Gibco, Thermo Fisher Scientific, Grand Island, NY, USA), and PC-3 and prostate myofibroblasts WYMP-1 (CLS Cell Lines Service, Eppelheim, Germany) were maintained in DMEM (Gibco, Thermo Fisher Scientific, Grand Island, NY, USA) supplemented with 10% FCS. All the cells were maintained at 37 °C and 5% CO_2_. The medium was changed every week, and the cells were passaged.

### 2.3. Assembloid Culture

Assembloid were generated by combining 1 × 10^4^ cells per cell line per well in UltraLow Attachment Plates (ULA) (Corning, Berlin, Germany) as follows: RWPE-1/RWPE-1/WYPM-1 (healthy), RWPE-1/WYMP-1/BPH-1 (BPH), RWPE-1/WYMP-1/PC-3 or -/DU-145 or -/LNCaP (all 1 × 10^4^ cells 1:1:1) in a cell line-specific medium mixture (1:1:1). After two days of culture in ULA plates, assembloids were transferred into CERO tubes (OMNI Life Science, Bremen, Germany) and cultivated for the next 5 days in a 3D-CERO incubator (OMNI Life Science) under rotating conditions. After this time, assembloids as well as the SN were collected for further analyses.

### 2.4. Serum Protein Analysis

We analyzed the concentrations of PSA and ILs 6 and 8 in the subjects’ serum and in the SN from the assembloid growth.

PSA serum analysis was performed for every subject within the 10-year study period at each sampling time using a solid-phase chemiluminescent immunometric assay kit with an Immulite 1000 immunoassay system (Siemens Healthcare GmbH, Erlangen, Germany) according to the manufacturer’s instructions. We analyzed the fPSAs for 11 PCa patients using the same kits, and PSA ranged between 3.8 and 10 ng/mL.

Interleukins IL-6 and -8 were analyzed for each subgroup (healthy *n* = 19; BPH *n* = 19; PCa *n* = 19) using a solid-phase chemiluminescent immunometric assay kit with an Immulite 1000 immunoassay system (Siemens Healthcare GmbH, Erlangen, Germany,) according to the manufacturer’s instructions.

Both ILs were also analyzed by in vitro experiments. After 2 days of growth in ULA and 5 days in a 3D-CERO incubator, the supernatant was collected and tested with the abovementioned tests. Cytokine levels were measured from pooled one-week supernatants (28.6 mL of medium volume in a 3D-Cero Tube) of 288 assembloids per condition. Each condition represented one pooled sample per experiment, and experiments were performed in triplicate (*n* = 3). No normalization to cell number, protein content, or assembloid size was applied.

### 2.5. miR Isolation and cDNA Preparation

Cell-free RNA was isolated from 200 µL of serum or 400 µL of assembloid SN using the miRNeasy Serum/Plasma Advanced Kit (Qiagen, Hilden, Germany) according to the manufacturer’s instructions. Briefly, protein complexes in the serum were lysed and denatured. All the contaminants were washed away, and the miRNA was eluted with water. Five microliters of isolated miR served as a template for cDNA synthesis, which was performed using the TaqMan Advanced miRNA cDNA Synthesis Kit that is specific for TaqMan Advanced miRNA Assays (both Thermo Fisher, Berlin, Germany).

### 2.6. Digital PCR (dPCR)

Digital PCR (dPCR) was performed using a QuantStudio Absolute Q digital PCR system (Thermo Fisher). A total reaction volume of 9 µL was loaded into the chambers of the Microfluidic Array Partitioning (MAP) plate and overlaid with isolation buffer (both Thermo Fisher). The PCR mixture consisted of 2 µL of Applied Biosystems™ Absolute Q™ DNA Digital PCR Master Mix, 0.5 µL of the TaqMan assay, and 7.5 µL of water or sample. The TaqMan assays used included hsa-miR-16-5p (Assay ID: 477860_mir), hsa-miR-141-3p (Assay ID: 478501_mir), hsa-miR-221-3p (Assay ID: 477981_mir), hsa-miR-222-3p (Assay ID: 477982_mir), and hsa-miR-375-3p (Assay ID: 478074_mir). The run started with 3 min of preheating at 95 °C, 5 s at 95 °C, and 40 cycles of 30 s at 62 °C. The copy numbers per microliter (cp/µL) were automatically calculated using QuantStudio Absolute Analysis Software (v: 6.2.0) (Thermo Fisher). No-template controls (NTCs) were included in each dPCR run to monitor for potential contamination. miRNA concentrations were quantified by absolute digital PCR and are reported as copies/µL. As all samples were processed under identical experimental conditions, performed from the same blood volume, direct comparison of absolute copy numbers was considered appropriate. No endogenous reference miRNA was used for normalization. Each in vitro experiment was performed in duplicate and repeated three times. Investigators were not blinded during the experimental procedures or data analysis.

### 2.7. Assembloid Staining

#### 2.7.1. H&E

All assembloids were cultured as described above. After 7 days, they were collected and washed with PBS (Sigma/Merck, Darmstadt, Germany), stained with hematoxylin for 1 min, and washed in tap water followed by distilled water. The assembloids were then fixed with 4% Roti-Histofix (Carl Roth, Karlsruhe, Germany) for 30 min, dehydrated for 15 min with 100% ethanol, collected in an Eppicap and covered with 2% agarose (PeqLab, Erlangen, Germany). Agarose blocks were fixed in 4% paraformaldehyde (PFA) and dehydrated with ethanol and embedded in paraffin.

The cut slides (4 µm thin) were deparaffinized and rehydrated, and stained with hematoxylin and eosin. Slides were subsequently dehydrated with increasing concentrations of alcohol and covered with mounting medium. Light microscopy images were obtained using a ZEISS Axio Observer microscope (Carl Zeiss Microscopy GmbH, Jena, Germany).

#### 2.7.2. Masson’s Trichrome Staining for Collagen

Masson’s Trichrome staining was performed at the Institute of Pathology, UMH. Deparaffinized and rehydrated slides were subsequently washed in distilled water and stained with an Artisan Masson’s Trich kit (Agilent Technologies, Waldbronn, Germany).

### 2.8. Statistical Analysis

GraphPad Prism version 9.0 (GraphPad Software, La Jolla, CA, USA) was used for all statistical analyses and figure generation. Data were tested for normality using the Shapiro–Wilk test. Depending on normality, group comparisons were performed using one-way ANOVA or the Kruskal–Wallis test. For nonparametric analyses, Dunn’s multiple-comparison post hoc test was applied, and adjusted *p*-values were reported for pairwise comparisons. To account for multiple testing across the investigated biomarkers, *p*-values from the global Kruskal–Wallis analyses were additionally adjusted using the Benjamini–Hochberg false discovery rate (FDR) procedure. Data are presented as median (range).

Diagnostic performance of miRNAs, interleukins, and PSA was evaluated using receiver operating characteristic (ROC) curve analysis in GraphPad Prism 9. Area under the curve (AUC) values with 95% confidence intervals and exact *p* values were calculated. Optimal cut-off values were determined using the Youden index, and the corresponding sensitivity and specificity were reported. No internal validation, like bootstrapping or cross-validation, was performed; therefore, ROC-based estimates were interpreted as exploratory.

Correlations between miRNAs and PSA levels were assessed using Spearman’s rank correlation coefficient and interpreted as weak (r = 0.1–0.3), moderate (r = 0.3–0.5), or strong (r = 0.5–1.0).

## 3. Results

### 3.1. Characterization of the Cohort

**Figure 1:**
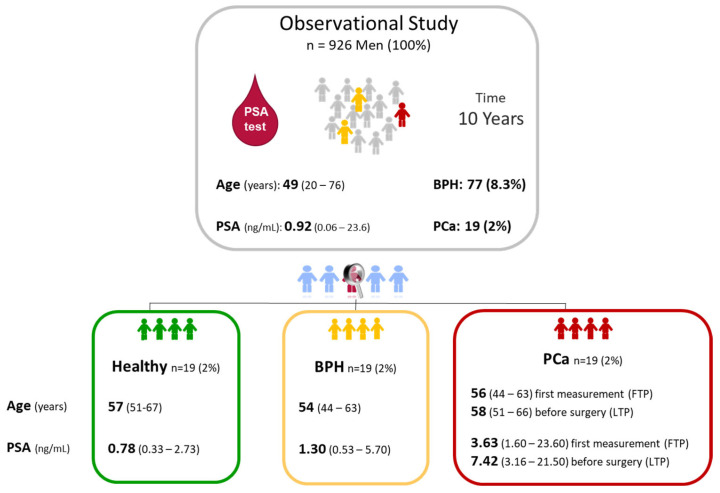
Graphical presentation of the participant cohort. Within the 10 years of the study, we analyzed PSA expression in 926 men. For further analysis, we included 19 men who developed PCa, 19 men with BPH, and 19 randomly chosen, age- and BMI-matched healthy men (exploratory cohort).

A total of 926 men employed by public utility companies or retired from these institutions participated in a 10-year observational study on prostate cancer. In accordance with the medical guidelines published in 2010, the recommended screening interval for PCa was 2–4 years. Shorter follow-up intervals were scheduled for participants with a family history of PCa (*n* = 69), high PSA levels, or a participant age that was younger than the recommended screening age. At the first visit, the median age of the participants was 49 (20–76) years, and BMI calculations indicated that they were overweight [27.9 kg/m^2^ (range, 17.9–45.7)]. In total, 238 participants reported taking medication. The level of PSA was 0.92 ng/mL (0.06–23.6) (Table 1). However, within the 10 years of the study, 19 men (2%) were diagnosed with PCa, and 77 men (8.3%) were diagnosed with BPH. We performed fPSA analysis in 11 PCa patients whose PSA concentration ranged from 3.8 to 10 ng/mL. Pretreatment samples from all PCa patients and 19 age- and BMI-matched BPH patients before initiating medication and samples from 19 healthy participants at the corresponding ages without a family history of the disease, were included for further analysis (Figure 1, Table 1).

Among the 19 PCa patients, 3 (16%) reported a family history of the disease. The median time from the first visit to the last visit before surgery was 2 years (range 0.0–9.0) or 100 weeks (6.0–495). At the beginning of the examination (FTP), the patients had a median age of 55 (44–63) years, with a median PSA level of 3.6 (1.6–23.6) ng/mL. At the last sample collection time before surgery (LTP), the median age was 58 (51–66) years, and the median PSA concentration was 6.34 (3.16–21.5) ng/mL. In patients suspected of PCa, with PSA between 3.8 and 10 ng/mL, fPSA was routinely measured as part of the standard clinical evaluation. The median percentage of fPSA/PSA was 10.7 (5.1–24.6) % (Table 2). Histopathological examination revealed a GS of 6–7 for 17 patients and 9 for one patient. The GS status of one patient was unavailable. No association between GS and PSA level or time from FTP and LTP was observed. Most of the tumors were stage T2 (*n* = 8), four were stage T3, and one was stage T1 (Table 3). One patient developed bone metastases 6 years after the initial diagnosis. In patients with a tumor of GS 9 and unknown status, the PSA concentration was greater than 10 ng/mL. Among the BPH patients, 6 (32%) reported a family history of PCa. At the first sampling time point, they had a median age of 54 years (44–63) and a lower median PSA concentration (1.30 ng/mL [0.53–5.7]). The median PSA concentration did not change significantly and reached 1.34 ng/mL (0.32–2.27) during the follow-up period (60 years [49–66]). Only pretreatment samples collected at the first time point were included in the biomarker analyses. The PSA results for the group of age-matched healthy men (52 [45–61] years) were within the normal range of 0.78 (0.28–2.7), and none of the participants had a family history of PCa (Table 2).

### 3.2. Assessment of PSA and miRs in the Cohort

**Figure 2:**
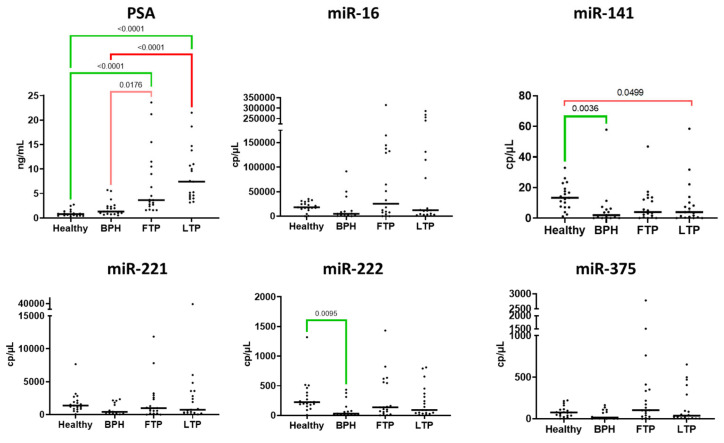
Serum expression of PSA (ng/mL) and miRs (cp/µL) of the exploratory cohort (each group *n* = 19). PSA levels were significantly higher in the PCa group than in the healthy and BPH groups. The levels of miR-222 and miR-141 were significantly lower in the BPH group than in the corresponding healthy control group.

As a standard biomarker, we analyzed the PSA concentration. Its levels were significantly higher in the PCa group at both sampling time points, FTP (3.630 [1.6–23.6]) and LTP (7.42 [3.16–21.5]), from healthy (0.78 [0.33–2.73]) and BPH-affected men (1.3 [0.53–5.7]), but we could not find any differences between the BPH and healthy groups (Figure 2). Furthermore, we analyzed the serum levels of five miRs (miR-16, -141, -221, -222, and -375).

Serum levels of miR-16 in samples from tumor patients of the exploratory cohort (each group *n* = 19) were initially (FTP) higher (median [range]; 25,382 cp/µL [79–314,673]) than those in healthy subjects (18,132 cp/µL [0.0–35,159]). These levels decreased over time, reaching 48% of the FTP shortly before surgery (LTP 12,190 cp/µL [944–286,193]). Compared with those in BPH patients, the miR-16 signal levels in both FTP and LTP patients were also elevated (4822 cp/µL [148–91,495]). The levels of miR-141 in the FTP (3.975 cp/µL [0–46.8]) and LTP (3.95 cp/µL [0–58.45]) groups reached approximately 30% of the healthy control level (13.33 cp/µL [1.15–32.91], the highest among all groups), whereas the level in the BPH group (1.975 cp/µL [0–57.83]) reached only 15%. At both measurement times, the amount of circulating miR-221 was greater in PCa patients (FTP: 981 cp/µL [0.275–11,854] and LTP: 739.5 cp/µL [24.7–39,552) than in BPH patients (406.1 cp/µL [37.95–2340]) but lower than that in healthy men (1365 [60.68–7664]). Changes induced by miR-222 followed the pattern of changes induced by miR-221. Compared with those in healthy men (222.5 cp/µL [6.8–1319]), the miR-222 levels in PCa patients were initially reduced to 136 cp/µL (0.575–1432) at the FTP and further decreased to 90.55 cp/µL (8.225–810.1) at the LTP but were still higher than those in BPH patients (28.55 cp/µL; 3.1–433.4). The difference in expression of miR-375 was greatest between patients with pathological conditions and healthy men. The level of miR-375 expression for BPH patients was 16% of that of healthy men (15.3 cp/µL [1.125–164.7] and 76.5 cp/µL [3.4–222.2], respectively). In PCa patients shortly before surgery (37.8 cp/µL [1.975–653.2]), the level of miR-375 was 2.47-fold greater than that in BPH patients, corresponding to 49% of the healthy controls. The level of miR-375 decreased in PCa patients, reaching only 37% of the FTP level at the LTP. Initially, the PCa patients did not differ markedly from the healthy men but were 6.7-fold higher compared with the BPH patients (Figure 2). These findings were obtained from an exploratory cohort and require validation in larger studies to confirm the diagnostic potential of these biomarkers. After FDR correction with the Benjamini–Hochberg, the significant differences remained for PSA (FDR < 0.0006), miR-141 (FDR = 0.011), and miR-222 (FDR = 0.036), whereas miR-221, miR-375, and miR-16 showed no significant differences (all FDR > 0.05).

### 3.3. Relationships Between PSA Levels and miR Levels

**Figure 3:**
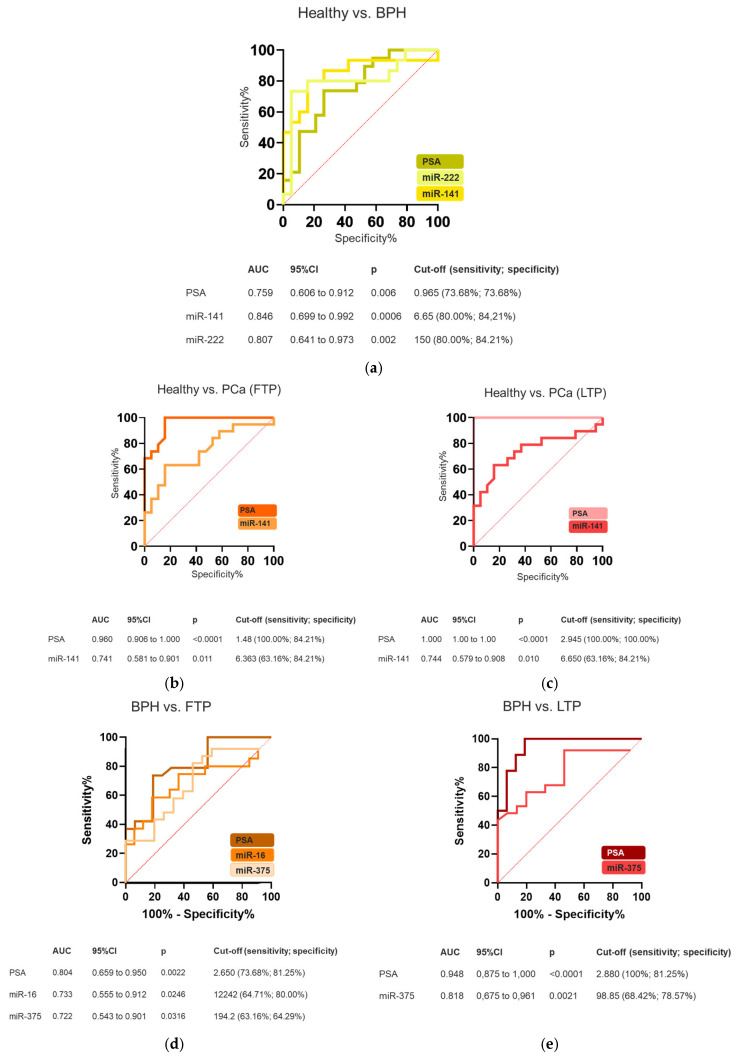
Receiver-Operation-Characteristics of the PSA and miR levels in the exploratory cohort. In the BPH group vs. the healthy group, four miRs had high diagnostic performance, with the diagnostic performance of miR-141 and miR-222 being even greater than that of PSA (**a**). In the PCa vs. healthy groups, at both FTP and LTP ((**b**,**c**), respectively), PSA showed the highest accuracy, followed by miR-141. Compared with the BPH group, the PCa group had the highest specificity and sensitivity for PSA (**d**,**e**). At each time point, FTP and LTP revealed additional indicators—miR-375 (FTP) and miR-16 (LTP) (**d**,**e**).

On the basis of the results of the PSA and miR expression analyses, we performed ROC curve analysis to differentiate the groups from each other. The question was whether we could identify factors differentiating the healthy, BPH, and PCa groups from each other (Figure 3).

Comparisons of the BPH and PCa groups to healthy men (exploratory cohort) revealed that the PSA level, especially among PCa patients, had the highest diagnostic performance, particularly in patients with PCa (AUC FTP: 0.960, 95% CI: 0.9 to 1.0; AUC LTP: 1.00, 95% CI: 1.0 to 1.0) (Figure 3b,c). In the BPH group, PSA (AUC: 0.759; 95% CI: 0.606 to 0.912) and two miRs, miR-16 (AUC: 0.709; 95% CI: 0.501 to 0.916) and -221 (AUC: 0.711; 95% CI: 0.517 to 0.904), had good/moderate performance, whereas miR-222 (AUC: 0.807; 95% CI: 0.641 to 0.973) and miR-141 (AUC: 0.846; 95% CI: 0.699 to 0.992) had very strong performance (Figure 3a). Compared with the healthy controls, miR-141 also showed moderate diagnostic performance at FTP (AUC: 0.741; 95% CI: 0.581 to 0.901) and LTP (AUC: 0.744; 95% CI: 0.579 to 0.908) (Figure 3b,c) and was the only miR that showed discriminatory ability between PCa vs. normal and BPH vs. normal samples.

When comparing patients with PCa and BPH, PSA remained the most accurate marker at both time points (FTP AUC: 0.804, 95% CI: 0.659–0.950; LTP AUC: 0.948, 95% CI: 0.875–1.000). Among the investigated miRNAs, miR-375 at FTP (AUC: 0.722, 95% CI: 0.543–0.901) and miR-16 at LTP (AUC: 0.689, 95% CI: 0.508–0.870) also demonstrated discriminatory performance for distinguishing PCa from BPH potential for PCa status (Figure 3d,e).

### 3.4. Assessment of Interleukins in the Cohort

We detected secreted IL-6 in 5 of 19 samples (26%) from PCa patients (both FTP and LTP), 5 of 19 samples (26%) from BPH patients, and 3 of 19 (16%) samples from healthy individuals. The median level of IL-6 at the FTP was 0.0 pg/mL (0.0–43.6), and the mean was 2.9 pg/mL. At LTP, the median concentration was still 0.0 pg/mL (0.0–12.7), but the mean concentration was 1.4 pg/mL. In the BPH group, IL-6 levels were comparable to those in the PCa group (0.0 pg/mL [0.0–3.8]), but the mean value was lower at 0.8 pg/mL. In the healthy group, the median concentration varied only slightly from that in the BPH patients, which was 0.0 pg/mL (0.0–4.8), with a mean of 0.5 pg/mL.

**Figure 4:**
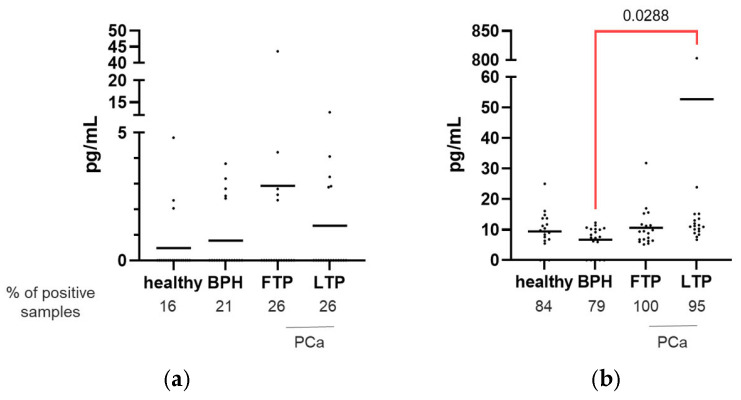
IL secretion levels in the cohort. Different secretion levels of IL-8 (**b**) but not IL-6 (**a**) were detected between BPH patients and PCa carcinoma patients shortly before surgery.

IL-8 was detected in all the PCa patients at the FTP (median 9.4 pg/mL [5.2–31.8]; mean 10.62 pg/mL) and in 18 of 19 (95%) at the LTP (median 11.0 pg/mL [0.0–803.0]; mean 52.7 pg/mL). IL-8 was present in 15 of 19 (79% BPH samples, with a median of 7.7 pg/mL (0.0–12.3), and the mean was 7.0 pg/mL. In healthy controls, IL-8 was detected in 16 of 19 samples (84%), with a median of 9.4 pg/mL (range, 0.0–25.0) and a mean of 9.4 pg/mL. (Figure 4b).

### 3.5. Correlations Among miRs and Between miRs and PSA

**Figure 5:**
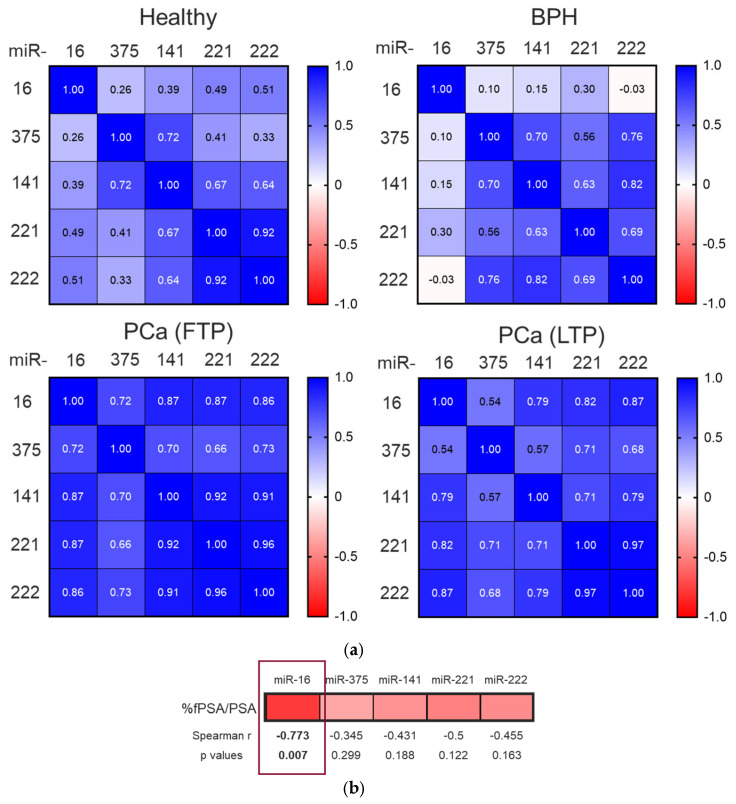
A heatmap displays the correlations between groups. Correlations between miR-16, miR-375, miR-141, miR-221, and miR-222 in the PCa group at both FTP and LTP were more frequent and stronger than those in the BPH and healthy groups of the cohort (**a**). Correlation between %fPSA/PSA and the analyzed miRs. %fPSA/PSA correlated negatively with miR-16 in the LTP PCa group (**b**). Spearman’s r values (r < 0—negative; r > 0—positive correlation).

Most miRs exhibited strong correlations with one another in PCa patients at FTP (*p* ≤ 0.002), involving all five analyzed miRs. At LTP, four miRs remained slightly reduced and correlated (r ≥ 0.539; *p* ≤ 0.017). At FTP, the strongest associations with other miRs were observed for miR-16 (*p* ≤ 0.001) and miR-141 (*p* ≤ 0.009). At LTP, miR-375 lost its correlation with miR-16 (r = 0.539; *p* = 0.017) and showed only a weak correlation with miR-141 (r = 0.573, *p* = 0.010), whereas miRs-221 and -222 remained strongly correlated (miR-221: r = 0.709, *p* = 0.001; miR-222: r = 0.677, *p* = 0.001). The healthy subject group showed strong and moderate correlation patterns, which were even weaker in the BPH subgroup (Figure 5a).

Analysis of the %fPSA/PSA revealed a strong negative correlation with miR-16 in the LTP PCa group (r = −0.773; *p* = 0.007) (Figure 5b).

### 3.6. Age-Dependent Expression of miRs and PSA

**Figure 6:**
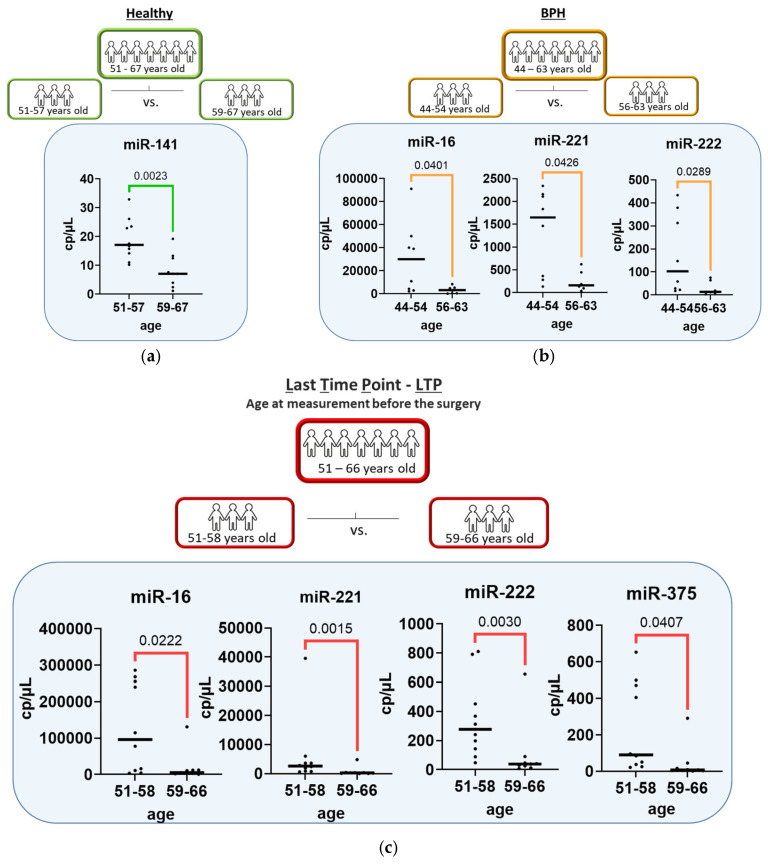
The expression of miRs depends on age. The healthy (green) (**a**), BPH (yellow) (**b**), and PCa (red) (**c**) groups were split into two subgroups (“younger” and “older”). Within the healthy group, only miR-141 expression was decreased in older individuals (**a**). In the BPH and PCa groups (**b**,**c**), the expression of miR-16, miR-221, and miR-222 decreased in the older group; additionally, in the PCa (LTP) group, the expression of miR-375 decreased in older patients (**c**).

To assess age-related differences in miR expression, each group was divided into “younger” and “older” subpopulations. Among the healthy controls, we observed significantly lower levels of miR-141 in the older subpopulation (age: 51–57 years; *n* = 10 vs. age: 59–67; *n* = 9) (Figure 6a). In the BPH group, younger men (age: 44–54 years; *n* = 9) showed significantly higher levels of miR-16, miR-221, and miR-222 than older men (age: 56–63 years; *n* = 7) did (Figure 6b).

For the PCa cohort, we performed two separate age-related analyses. First, patients were grouped according to age at the first sampling time (FTP; 44–63 years) into younger (44–56 years, *n* = 10) and older (57–63 years, *n* = 9) subgroups. In this analysis, four of the five miRs (miR-16, miR-221, miR-222, and miR-375) were expressed at lower levels in older patients exclusively at the time point shortly before surgery (LTP) but not at the FTP. Second, patients were stratified by age at LTP (51–66 years) into younger (51–58 years, *n* = 10) and older (59–66 years, *n* = 9) groups. This analysis revealed similar results, with significantly reduced levels of the same miRs (miR-16, miR-221, miR-222, and miR-375) in older patients at LTP (Figure 6c).

We performed the same analysis for PSA, but no significant age-related differences were observed.

### 3.7. In Vitro Model

Six prostate cell lines were combined to generate 3D prostate assembloids. Healthy cells (RWPE-1), stromal fibroblasts (WPMY-1), BPH (BPH-1), and one of the three PCa cell lines, LNCaP, DU-145, or PC-3, were cultivated as assembloids under stable (ULA-plate) conditions, followed by rotation (3D-Cero-incubation) (Table 4).

### 3.8. Histo/Morphologic Characterization of Assembloids

**Figure 7:**
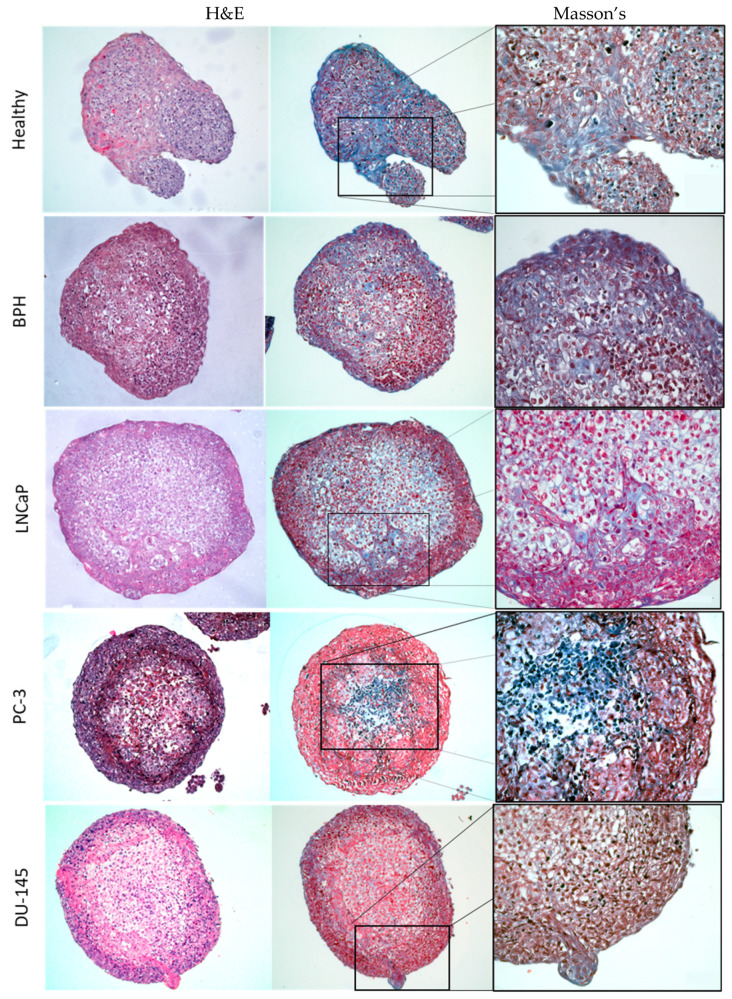
Three-dimensional assembloid model of prostate cells. Representative H&E (**left**) and Masson’s trichrome (**middle**) staining of assembloids acquired with a 20× objective. The boxed area is shown at 40× magnification (**right**) to highlight the cellular morphology and extracellular matrix (ECM) organization.

The heterogeneous structure of the assembloids was confirmed by Mayer’s hematoxylin and Masson’s staining, with all the examined assembloids exhibiting regions of diverse morphologies (Figure 7).

Healthy assembloids displayed typical cell area organization. They showed regions with dense aggregates of cells with round/oval nuclei, which indicated epithelial cells (RWPE-1), and regions with loose, spindle-shaped areas and elongated cell nuclei, which were typical of fibroblasts (WPMY-1), corresponding to the tissue stroma. Additionally, Masson’s Trichrome staining confirmed collagen deposition within stromal areas. With respect to BPH assembloids, the cells secreting collagen in the stromal area were denser compared with those in healthy assembloids. The epithelial compartments were included as compressed cell nests between fibroblasts. Generally, the architecture displayed greater mixing of epithelium-like and stroma-like areas, which could correspond to BPH tissue. The organization of tumor assembloids differed from that of healthy assembloids. In LNCaP assembloids, solid epithelial-like regions surrounded stromal, collagen-producing cells. A similar pattern was observed for the DU-145 assembloids; however, the ECM reorganization showed continued production of collagen and epithelial-like outer regions interwoven with collagen-rich cells. PC-3 assembloids lacked this organization, exhibiting a domination of epithelial-like cells instead and loosening in the central region with less organized, reduced collagen.

(a) Characterization of miRs in Assembloids

**Figure 8:**
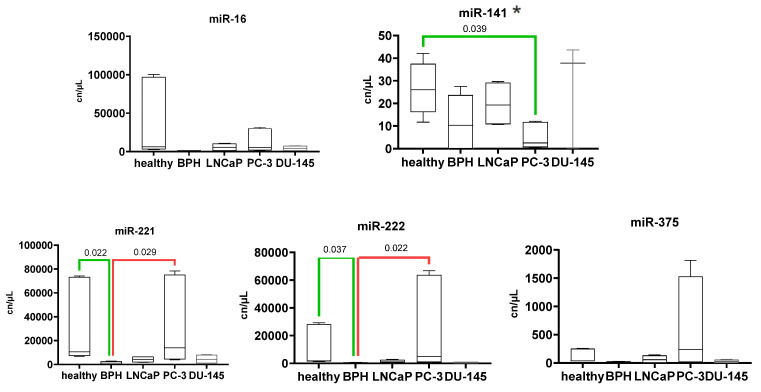
Secretion of miRs in the supernatants of assembloids. The levels of miR-222, -221, and -141 were significantly lower in the BPH group than in the corresponding healthy control group. Additionally, compared with healthy assembloids, PC-3 assembloids secreted less miR-141; * FDR negative.

The secretion of three of the five analyzed miRs significantly differed between the assembloid models. The median levels of miR-16 in samples from tumor assembloids were slightly reduced compared with those in healthy assembloids (6379 cp/µL [2690–100,277]): 0.90-fold in LNCaP (5711 cp/µL [1250–10,782]) and 0.81-fold in PC-3 (5139 cp/µL [1504–31,526]) but only 0.22-fold in DU-145 (4193 cp/µL [990.8–7639]). Compared with the BPH-assembloids (1395 cp/µL [56.92–1737]), the healthy ones had values that were 4.57-fold higher: LNCaP: 4.9-fold higher; PC-3: 3.68-fold higher; and DU-145: 3-fold higher. We found the strongest suppression of miR-141 levels by PC-3 assembloids (90%; 2.490 [0.43–1206]) followed by BPH assembloids (60%; 10.32 [0–27.47]), when LNCaP reached 74% (19.27 [10.66–29.66]) of healthy control (26.07 [11.68–42.12]) and DU-145 was 1.45-fold higher (37.77 [0–43.66]). The lowest secretion of miR-221 was detected in BPH assembloids (2266 cp/µL [192.7–2709]), which was significantly (40-fold) lower than that in healthy assembloids (10,580 cp/µL [6701–74,299]). In addition, the levels of miR-221 sn in assembloids containing PCa cells were higher than those in BPH cells: LNCaP cells: 1.87-fold (4249 cp/µL [1782–6638]); PC-3 cells: 6.18-fold (14,005 cp/µL [3903–78,302]); and DU-145 cells: 1.90-fold (4309 cp/µL [933.2–8184]). The highest secretion was observed for the PC-3 assembloids. The secretion pattern of miR-222 reflected the signature of miR-221. In addition, in this case, the lowest level was observed for the BPH assembloid (414.9 cp/µL [22.64–659.0]), which was 4.01-fold lower than that in the healthy group, when LNCaP was 2.64-fold higher (1115 cp/µL [283.6–2851]), PC-3: 11.6-fold (4881 cp/µL [828.7–66,748]) and DU-145: 1.31-fold higher (550.7 cp/µL [67.94–1116]). Like miR-221, PC-3 assembloids produced the highest level of miR-222. The BPH assembloids exhibited almost complete loss of miR-375 secretion (only 1,130 cp/µL [0–29.74]), whereas PC-3 (237.7 cp/µL [11.32–1817]) assembloids displayed a strong increase, exceeding the healthy controls (38.30 cp/µL [32.26–259.9]) by more than 6-fold and the BPH assembloids by 210.5-fold (Figure 8). After Benjamini–Hochberg FDR correction, miR-221 (FDR = 0.017) and miR-222 (FDR = 0.017) remained below the significance threshold. The observed effect for miR-141 was no longer evident after correction 0.58 (FDR = 0.051). miR-375 and miR-16 did not meet the threshold (both FDR > 0.05).

(b) Assessment of Interleukins in the Assembloids

**Figure 9:**
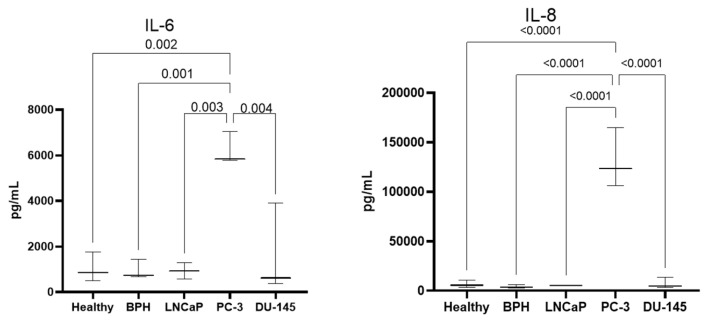
Secretion of IL-6 (pg/mL) and IL-8 (pg/mL) by assembloids. Compared with all the other assembloids, mesenchymal, bone metastatic assembloid PC-3 secreted increased levels of both interleukins.

IL-6 and IL-8 concentrations were measured in pooled one-week supernatants (28.6 mL) obtained from 288 assembloids per condition (Figure 9). PC-3 assembloids exhibited the highest secretion of both cytokines. Mean IL-6 levels were approximately sixfold higher than those of healthy assembloids, whereas IL-8 concentrations were increased by approximately 20 pg/mL (mean) and 12 pg/mL (median). Compared with the other PCa assembloid models, particularly LNCaP, PC-3 assembloids consistently secreted higher levels of IL-6 and IL-8, exceeding LNCaP by at least 7 pg/mL (mean) and 6 pg/mL (median) for IL-6, and by 32 pg/mL (mean) and 24 pg/mL (median) for IL-8.

## 4. Discussion

In accordance with the guidelines for PCa prevention, PSA measurements should be offered to 45-year-old men with a life expectancy of at least 10 years [32]. We describe a ten-year observation study for PCa prevention based on PSA or PSA and fPSA measurements that was performed in our clinic with 926 participants working for (or pensioners of) public utility companies. During this 10-year period, we diagnosed PCa, which was usually of clinically insignificant status, in 2% of the examined men and BPH in approximately 8% of the men. The long-term study provided the possibility of observing increases in PSA levels over time in some participants and diagnosing them. Elevated PSA levels in PCa patients at both sampling time points confirmed the presence of active tumor epithelial compartments. These findings highlight the importance of early detection strategies. The PROBASE study revealed that compared with digital rectal examination, PSA testing for PCa prevention is more efficient [33]. Ilic et al. [34] reported an increase in the detection of low-stage cancers after PSA screening. In contrast, other investigations have confirmed that the analysis of PSA levels is an important part of prophylactic examination, as it is an indication of cancer suspicion, but its use alone could lead to overdiagnosis [35]. One factor that improves validation is the fPSA/PSA ratio, which is routinely determined in patients with PSA between 3.8 and 10 ng/mL. Recently, Ferraro et al. described a nomogram based on the combined calculation of factors such as the PSA level, fPSA level, and age, which can estimate the risk of high-grade or low-grade PCa and aid in the decision-making process. With the use of a nomogram, they were able to discriminate between high-grade tumors and low-grade tumors or no disease [36]. In our study, almost all the diagnosed cancers were at low/intermediate risk [32] stage (PSA > 3 ng/mL, %f/PSA 5.1–24.6), and even if all the therapeutic options were presented to the patients, they decided to undergo radical prostatovesiculectomy. To critically address the aspect of overdiagnosis, we analyzed other factors, such as cytokines and miRs, which are known to play a role in advanced, metastatic tumors. We performed LB analysis for IL-6 and IL-8 and 5 miRs in the exploratory cohort (each subgroup *n* = 19) of healthy individuals and BPH and PCa patients diagnosed during our studies. To better understand biomarker alterations associated with benign prostate enlargement and inflammatory processes, we compared patients with BPH and healthy controls. These analyses were primarily intended to provide biological context rather than to demonstrate clinical diagnostic utility. Moreover, to investigate the role of miRs in prostate diseases, we generated in vitro assembloids representing healthy, BPH, and different PCa cell lines. Analysis of serum PSA and circulating miRs revealed distinct molecular differences between PCa, BPH, and healthy individuals, which could be partially recapitulated by the biological characteristics observed in the assembloid models. The present findings were compared with published data regarding cytokine and miR expression in patients with advanced PCa and with the molecular characteristics observed in the in vitro assembloid models.

We detected low serum IL-6 levels in samples from healthy, BPH, and PCa patients in our cohort. The frequency of detected IL-6 secretion was greater in PCa patients than in BPH patients (5% detection rate) and healthy subjects (10% detection rate). This distribution is consistent with the low stage of PCa in patients. Multiple findings implicate IL-6 in the invasive behavior of cells, suggesting its role in antiapoptotic or proepithelial–mesenchymal-transition processes [37]. IL-6 is known to be a surrogate marker of metastatic and Castration-Resistant Prostate Cancer patients [8]. Our data revealed that IL-6 was detectable in only a minority of serum samples and at low concentrations. Its expression appeared to be patient-specific, indicating that IL-6 should be considered as an acute individual inflammatory mediator rather than a low-stage tumor or progression marker. Although our in vitro assembloid model demonstrated an association between IL-6 secretion and aggressive, mesenchymal, and androgen-independent PCa (PC-3), it showed limited diagnostic utility in the present exploratory cohort. IL-8 is also involved in many tumor-associated processes, such as inflammation and angiogenesis, and it is well known to be involved in the EMT, invasion, and metastasis of PCa [38,39]. In our exploratory cohort, detectable IL-8 was observed in nearly all PCa patients, whereas it was less frequent in healthy controls and BPH patients. A markedly increased IL-8 concentration was detected in only one PCa patient before surgery, and therefore, this observation should be interpreted with caution. Consistent with this observation, the highest secretion of IL-8 was detected in the PC-3 assembloid model, which represents an aggressive, androgen-independent prostate cancer phenotype. Although both cytokines are involved in the pathobiology of PCa, neither IL-6 nor IL-8 alone can serve as a definitive diagnostic marker for PCa. Their values are best interpreted in the context of tumor stage and other clinical parameters.

To provide the biological context for the changes observed in the PCa group, we also compared the BPH and healthy individuals. The reduction in several circulating miRs in BPH patients compared with that in both healthy controls and PCa patients suggests a reduced epithelial turnover or altered secretory dynamics rather than malignant transformation. In contrast, PCa patients retained intermediate or partially preserved miR secretion levels. A Similar pattern was also observed in the structural organization of assembloids (Figure 7), where epithelial cells form the outer cellular layer and represent the primary secretory interface toward the surrounding microenvironment. This gives a perspectival opportunity to further in vitro studies of molecular changes taking place in such circumstances.

miRs are considered diagnostic and prognostic biomarkers that can be combined with PSA. Many investigators suggest the detection of these molecules, especially in the circulating form in LB, as PCa biomarkers [21]. As previously described, the %fPSA/PSA is a useful factor for the discrimination of high-grade tumors. However, Ferraro et al. reported that nomograms based on, among others, fPSA and PSA levels are associated with a risk of underestimation of predictive probabilities [36]. In our exploratory study, we observed a negative correlation between the fPSA/PSA ratio and secreted miR-16 in patients with PCa. As the free-to-total PSA ratio is primarily used in men with suspected prostate cancer to improve the specificity of PSA testing and guide biopsy decisions, the observed association may be of potential interest. However, due to the limited sample size and the lack of control groups in this analysis, these findings should be considered preliminary and hypothesis-generating. Further studies in larger, well-characterized cohorts, including benign prostatic hyperplasia and healthy controls, are needed.

miR-141 and miR-221/-222 are known to function as oncogenes or tumor suppressor genes, depending on the biological context. In our studies, these miRs showed disease-dependent regulation patterns, with the greatest reductions observed in BPH samples. The relatively preserved secretion in PCa patients compared with that in BPH patients may indicate increased epithelial turnover in malignant tissue. In vitro analysis of the PC-3 and DU-145 assembloids, representing advanced, poorly differentiated, androgen-independent prostate cancer phenotypes, revealed distinct cellular architectures and reduced secretion of miR-221/222 compared with RWPE assembloids. These cell lines were selected as well-established models of advanced prostate cancer to investigate tumor–stroma interactions and disease-associated biomarker secretion under controlled experimental conditions. Therefore, the assembloid experiments provide mechanistic insight into molecular alterations but should not be interpreted as a direct model of the predominantly localized prostate cancers included in the clinical cohort. These experimental findings complement the clinical observations by providing a biological context rather than direct validation of the serum biomarker findings.

As previously mentioned, the function of a miR depends on its binding site [12,13,40]. Several studies have reported that circulating miR-141 levels are higher in metastatic PCa patients than in healthy controls [19] and are increased in high-risk compared with intermediate-risk patients [23] whereas lower levels have been observed in localized patients than in healthy men [41]. It is proposed to be an onco-mir that responds to androgen [42] and is a metastasis marker of PCa [5]. In contrast, Luo et al. [43] associated miR-141 expression with reduced aggressiveness of PCa cells, Zhang et al. [44] reported lower levels of this miR in BPH patients (not significantly different) than in local or metastatic PCa patients, supporting its potential diagnostic and prognostic value [43]. Liu et al. [45] described the tumor- and metastasis-suppressive effects of miR-141 in PCa stem cells. Consistent with these findings, Huang et al. showed that the expression of miR-141 could suppress an epithelial–mesenchymal transition, invasion, and bone metastasis in PCa. [46]. In our cohort, all tumors were diagnosed at a localized stage, whereas the assembloids were created from metastatic PCa cell lines. We detected rather low serum levels of miR-141, especially in the BPH group, followed by the PCa group. Additionally, the level of miR-141 decreased in “older” healthy men, suggesting tissue reprogramming. To explain the issue of pathological samples, we analyzed the supernatants of the assembloids. We demonstrated that only the bone metastatic cell line PC-3 assembloid (mesenchymal and aggressive cell line) significantly reduced the secretion of miR-141. These data support the suppressive role of miR-141 in the aggressive behavior of PCa targeted to bones rather than in the development of tumors. Accordingly, ROC analysis suggests that miR-141 may provide complementary diagnostic information to PSA.

Additional regulated miRs identified in our studies were miR-221 and -222. In the literature, the expression of these miRs is synchronous [47]. In our study, both miRs showed similar expression patterns and strong diagnostic performance, consistent with previous reports demonstrating their value in combination with other PCa biomarkers [48]. miR-222 is involved in EMT and invasion by PCa [43,49]. Consistent with these observations, our assembloid model showed increased secretion of miR-222 in aggressive, mesenchymal, androgen-independent tumors, such as PC-3, consistent with its reported association with advanced disease. The literature provides evidence that AR represses miR-221/-222 expression [47]. In androgen-independent PCa, however, miR-221/-222 are upregulated, promoting cell proliferation, migration, and invasion. Interestingly, androgen-independent cells can release exosomes containing miR-222, which can be taken up by cells in androgen-dependent PCa and, in this way, increase the aggressive behavior of androgen-dependent cells [50]. In contrast, knockout of miR-222 enhances the proliferative and invasive abilities of (androgen-dependent) LNCaP cells, suggesting that it has tumor suppressor activity in these cells [51]. Gui et al. [47] analyzed the relationship between AR expression and miR-221/222 status. They concluded that the oncogenic function of those miRs is associated with AR expression and is transient, since the reactivation of AR-mediated pathways in previously negative cells may support the growth of PCa cells [47]. We did not determine the AR status of our patients; however, the low expression of miR-222 in BPH patients and its attenuation in PCa patients led us to hypothesize that the AR level is higher in these patients. Moreover, BPH results from the accumulation of stromal and epithelial cells. Considering the role of miR-222 in suppressing proliferation, its decreased circulating levels in BPH patients may contribute to the pathological growth of the prostate gland. Among the investigated miRs, miR-375 showed the greatest difference between pathological and healthy conditions. Serum levels remained higher in PCa patients shortly before surgery than in BPH patients, suggesting persistent tumor-derived epithelial signaling. Increased levels of miR-375 in PCa exosomes have been reported by Gan et al. [52]. Within assembloids, differences in epithelial organization and differentiation state between androgen-sensitive LNCaP and androgen-independent PC-3 or DU-145 models could similarly influence miR secretion patterns, emphasizing the importance of epithelial identity as a determinant of extracellular communication. Selth et al. [53] analyzed the control of prostatic cell plasticity by miR-375. They reported a negative correlation between miR-375 and EMT signatures, highlighting mesenchymal–epithelial transition, which ultimately decreased the migration process.

Aging is accompanied by a chronic low-grade inflammatory state termed “inflammaging” and contributes to systemic alterations in intercellular communication, which thereby modify extracellular vesicle release, cargo composition, and circulating miR availability and stability. Aging-induced downregulation of miRs is also associated with other pathophysiological conditions, such as age-associated cardiac dysfunction [54] or ischemic brain injury [55]. miRs are involved in the immune response by regulating immune system factors [56]. Furthermore, reduced miR expression has been linked to multiple sclerosis, a neuroinflammatory disease [57]. Advanced age is also a risk factor for prostate diseases, since the probability of prostate tumors as well as BPH disease increases with age. Our results revealed the downregulation of miRs in older individuals. The pattern of miR levels in our cohort suggested the presence of differences in aging between physiological and pathological conditions. The levels of miR-141 decreased only in older healthy individuals, whereas the levels of the other tested miRs (miR-16, miR-221, miR-222, and miR-375) significantly decreased in both BPH patients and PCa patients. In aged healthy mice, the inhibition of miR-141 expression reduced inflammation features [58]. Moreover, it improved musculoskeletal health, as manifested by increased muscle fiber size and bone mineral density and volume [58]. The authors hypothesized that miR-141 may influence premature senescence and that reducing it can prevent this process. The decrease in miR-141 observed in older healthy individuals, together with its stable expression in patient samples, suggests that alterations in physiological miR-141 regulation may contribute to disease development. Likewise, miR-16 has been implicated in the induction of cancer cell senescence. There is evidence that miR-16 can potentially induce the senescence of cancer cells [59]. Reducing the levels of this miR may be a molecular response to avoid cellular senescence-associated tumor suppression and facilitate disease progression. The senescence process is dysfunctional in the pathophysiology of many age-related diseases. Vascular and metabolic diseases often accompany aging. Serum levels of miR-221/miR-222 are known to be decreased in response to these illnesses [24,60]. The miR-221/miR-222 cluster is also involved in the regulation of the immune response, and downregulation of both genes was observed after inflammation stimulation [24]. Our results revealed reduced expression of miR-375 in the older PCa group. To our knowledge, there is no information regarding the influence of aging on the expression of miR-375.

We correlated the expression of the miRs with each other within each group of the cohort. Considering that most miRs in blood samples are not organ-specific and that the assessment of one cancer-specific molecule has limited value, we propose an integrated calculation of the correlations among the three miRs as a supporting tool in the diagnosis of prostate disorders. Taken together, the exploratory cohort data and assembloid observations support the concept that epithelial architecture and differentiation status strongly influence secretory biomarker profiles. While BPH tissue exhibits reduced circulating miR levels consistent with nonmalignant remodeling, PCa-associated epithelial compartments maintain active molecular communication with the systemic environment. Therefore, assembloid systems represent a biologically relevant platform to model disease-specific secretion dynamics and may help to mechanistically interpret circulating biomarker signatures.

## 5. Limitations

Our study was based on serum samples collected within a 10-year observational cohort. Because of the limited available volume of serum after centrifugation, the number of biomarker analyses that could be performed was restricted. During the study period, 19 participants were diagnosed with PCa and were compared with 19 age- and BMI-matched BPH patients and 19 healthy controls. Although all PCa cases identified during follow-up were included, the relatively small exploratory cohort limits the statistical power of the analyses. Therefore, validation in larger, independent, preferably multicenter cohorts is warranted.

The objective of our study was to investigate the diagnostic potential of selected circulating miRNAs rather than to establish predictive models. Consequently, the statistical analyses were limited to group comparisons, correlation analyses, and ROC analyses. No multivariable regression analysis was performed. Although the cohort groups were matched for age and BMI, additional factors, including medication, inflammatory conditions, prostatitis, and tumor stage, may have influenced circulating biomarker levels.

Not all PCa surgeries were performed at our institution. For that reason, tumor tissue, complete pathological information, and long-term follow-up data were unavailable for a proportion of patients, limiting clinicopathological correlations.

Technical replicates were performed for all molecular assays according to the manufacturer’s protocols. No external spike-in controls, extraction controls, or hemolysis assessment were included. This should be considered a methodological limitation, particularly for circulating miRNA analysis.

Finally, the assembloid model represents an experimental in vitro model based on established PCa cell lines and does not fully recapitulate the biological complexity of localized prostate cancer. Nevertheless, it provides a valuable platform for investigating disease-associated molecular alterations under controlled experimental conditions. All samples were processed using standardized protocols under identical experimental conditions, supporting robust absolute miRNA quantification by digital PCR.

## 6. Conclusions

In this 10-year observational study including 926 participants, BPH was detected in 8.3% and PCa in 2.0% of the cohort. Our findings confirm the established diagnostic value of PSA for the detection of prostate disorders and demonstrate disease-specific alterations in circulating miRNA levels associated with prostate pathology and patient age. We established prostate assembloids representing distinct disease phenotypes, providing mechanistic insight into molecular alterations associated with PCa progression. Assembloids consisting of mesenchymal, bone metastatic cell line exhibited elevated IL-6 and IL-8 secretion together with reduced miR-141 and increased miR-221/222 expression, consistent with an aggressive tumor phenotype.

These findings should be interpreted in the context of the experimental model, as the assembloids were generated from advanced PCa cell lines and provide mechanistic rather than direct clinical evidence. From a clinical perspective, PSA remains the standard biomarker for the early detection and monitoring of PCa. However, our results suggest that circulating miR-141, miR-222, IL-6, and IL-8 may provide complementary biological information alongside PSA and could contribute to a more comprehensive characterization of prostate disease; however, the role of miR-141 in this context requires further clarification. Although the present study did not evaluate a combined diagnostic model, the integration of established biomarkers with circulating miRNAs and cytokines represents a promising strategy that warrants further investigation and validation in larger, independent cohorts.

## Figures and Tables

**Table 1:** Demographic characteristics of the study cohort. The whole cohort included 926 men.

Observational Study Cohort	
Whole study cohort (*n*)	926
Age years; median (range)	49 (20–76)
PSA ng/mL; median (range)	0.92 (0.06–23.6)
BMI; median (range)	27.9 (17.9–45.7)
BPH (*n*)	77
PCa family history (*n*)	69
Medication against (*n*)	
Hypertension	166
Diabetes	25
Cholesterol-lowering drugs	39
Thyroid	23
BPH	6
Blood thinner	25
Other tumors	2

**Table 2:** Specific descriptions of the exploratory cohort. Men who developed PCa (red) or BPH (yellow), as well as healthy men (green), are presented with the respective colors. * *n* = 11.

Characteristic	Healthy Controls (*n* = 19)	BPH (*n* = 19)	PCa (*n* = 19)
Age, years (FTP), median (range)	52 (45–61)	54 (44–63)	55 (44–63)
Age, years (LTP), median (range)	–	60 (49–66)	58 (51–66)
Family history of PCa, *n*	Unknown	3	3
PSA, ng/mL (FTP), median (range)	0.78 (0.28–2.70)	1.30 (0.53–5.70)	3.60 (1.60–23.60)
PSA, ng/mL (LTP), median (range)	–	1.34 (0.32–2.79)	6.34 (3.20–21.50)
fPSA/PSA ratio (%), median (range) *	–	–	10.7 (5.1–24.6)
BMI, kg/m^2^, median (range)	27.4 (23.0–34.3)	26.0 (23.0–33.3)	25.8 (23.1–32.1)
Antihypertensive medication, *n*	0	0	2
Antidiabetic medication, *n*	0	0	1

**Table 3:** Clinicopathological characteristics of PCa cohort.

Clinicopathological Characteristics of PCa Cohort								
Gleason score (*n*)			TNM Classification (*n*)			Grading (*n*)		
	6 (3 + 3)	3	pT:	T1	1		G1a	1
	7a (3 + 4)	8		T2	7		G2	4
	7b (4 + 3)	6		T3a	4		G3	3
	9 (4 + 5)	1		T3b	1			
	Not specified	1	pN:	N0	6			
UICC Staging (*n*)				N1	2			
	I	1		Nx	2			
	II	4	pM:	M0	5			
	III	2		Mx	3			
	IV	1	Not known		6			
	Not specified	11						

**Table 4:** Composition of assembloids. All assembloids were composed of three cell lines.

Assembloids	Cell Lines					
	RWPE-1 (Healthy)	BPH-1 (BPH)	WPMY (Fibroblasts)	LNCaP (mPCa-LN)	PC-3 (mPCa–Bone)	DU-141 (mPCa–Brain)
Healthy	++		+			
BPH	+	+	+			
PCa-LNCaP	+		+	+		
PCa-PC-3	+		+		+	
PCa-DU-145	+		+			+

+, standard cell number; ++, twofold increased cell number compared with the other cell types in the respective assembloid.

## Data Availability

The original contributions presented in this study are included in the article. Further inquiries can be directed to the corresponding author.
